# Supplementary material for: Long Non-Coding RNA KCNQ1OT1 Regulates Protein Kinase CK2 Via miR-760 in Senescence and Calorie Restriction
Source: Int J Mol Sci. 2022 Feb 8;23(3):1888. doi: 10.3390/ijms23031888 (PMC8836653; doi:10.3390/ijms23031888)
Supplement: Supplementary file 1 [file ijms-23-01888-s001.zip › Supplementary Figure legends.pdf]

Supplementary figure legends

Figure S1. The sequences and binding sites of lncRNA KCNQ1OT1, miR-760, and CK2α mRNA, which were determined with TargetScan and miRanda. SD, silencing domain; LINE, long interspersed element; ORF, open reading frame.
