# Supplementary material for: Long Non-Coding RNA KCNQ1OT1 Regulates Protein Kinase CK2 Via miR-760 in Senescence and Calorie Restriction
Source: Int J Mol Sci. 2022 Feb 8;23(3):1888. doi: 10.3390/ijms23031888 (PMC8836653; doi:10.3390/ijms23031888)
Supplement: Supplementary file 1 [file ijms-23-01888-s001.zip › Supplementary Table S1.pdf]

Supplementary Table S1. Sequences of miR-760 mimic and inhibitor used in this study.

miR-760 mimic	Forward: 5'-CGGCUCUGGGUCUGUGGGGA-3‘
	Reverse: 5'-UCCCACAGACCCAGAGCCG-3'
negative control	Forward: 5'-ACGUGACACGUUCGGAGAAUU-3'
	Reverse: 5'-UUCUCCGAACGUGUCACGUUU-3'
miR-760 inhibitor	RRRQRRKKRR-OO-TCCCCACAGACCCAGAGCCG
negative control	RRRQRRKKRR-OO-CTCCCTTCAATC

RRRQRRKKRR, cell penetrating peptide; OO, AEEA linker.
