# Supplementary material for: Long Non-Coding RNA KCNQ1OT1 Regulates Protein Kinase CK2 Via miR-760 in Senescence and Calorie Restriction
Source: Int J Mol Sci. 2022 Feb 8;23(3):1888. doi: 10.3390/ijms23031888 (PMC8836653; doi:10.3390/ijms23031888)
Supplement: Supplementary file 1 [file ijms-23-01888-s001.zip › Supplementary Table S2.pdf]

Supplementary Table S2. Primers used for RT-PCR analyses

Gene		Primer sequence
KCNQ1OT1-1	Forward	5′-CACTTCCAGTCCCACACCCT-3′
	Reverse	5′-CGCTGAGAACCACTCATTCA-3′
KCNQ1OT1-2	Forward	5′-AGCAGCCAGAAGGATGAGAA-3′
	Reverse	5′-ACCAGAAGGCAGAATGATGG-3′
CK2α	Forward	5′-AAGACCCTGTGTCACGAACCC-3′
	Reverse	5′-GGCTCCTCCCGAAAGATCATAC-3′
IL1β	Forward	5′-AGTGGCAATGAGGATGACTTGT-3′
	Reverse	5′-AGATGAAGGGAAAGAAGGTGCT-3′
IL6	Forward	5′-CACACAGACAGCCACTCACC-3′
	Reverse	5′-AGGTTGTTTTCTGCCAGTGC-3′
MMP3	Forward	5′-CTCACAGACCTGACTCGGTT-3′
	Reverse	5′-CACGCCTGAAGGAAGAGATG-3′
β-actin	Forward	5′-TCCCTGGAGAAGAGCTACGA-3′
	Reverse	5′-AGCACTGTGTTGGCGTACAG-3′
